# Apoptotic and anti-metastatic effects of the whole skin of *Venenum bufonis* in A549 human lung cancer cells

**DOI:** 10.3892/ijo.2011.1310

**Published:** 2011-12-20

**Authors:** JEONG-SEOK PARK, DONG YEOK SHIN, YEON-WEOL LEE, CHONG-KWAN CHO, GI YOUNG KIM, WUN-JAE KIM, HWA-SEUNG YOO, YUNG HYUN CHOI

**Affiliations:** 1 Department of East-West Cancer Center, College of Oriental Medicine, Daejeon University, Daejeon 301-724; 2 Department of Biochemistry, Dongeui University College of Oriental Medicine, Busan 614-052; 3 Department of Marine Life Sciences, Jeju National University, Jeju 690-756; 4 Department of Urology, Chungbuk National University College of Medicine, Cheongju 361-763; 5 Departments of Biomaterial Control (BK21 program), Blue-Bio Industry RIC, Dongeui University, Busan 614-714, Republic of Korea

**Keywords:** *Venenum bufonis*, A549 cells, apoptosis, invasion

## Abstract

In the present study, the effects of the whole skin of *Venenum bufonis* on apoptotic and anti-invasive activity in A549 human lung cancer cells were investigated. Treatment with extract of the whole skin of *V. bufonis* (SVB) resulted in a significant decrease in cell growth of A549 cells, depending on dosage, which was associated with apoptosis induction, as proved by chromatin condensation and accumulation of apoptotic fraction. SVB treatment induced expression of death receptor-related proteins, such as death receptor 4, which further triggered activation of caspase-8 and cleavage of Bid. In addition, the increase in apoptosis by SVB treatment was correlated with dysfunction of mitochondria, activation of caspase-9 and -3, downregulation of IAP family proteins, such as XIAP, cIAP-1 and cIAP-2, and concomitant degradation of activated caspase-3-specific target proteins, such as poly (ADP-ribose) polymerase and β-catenin proteins. However, z-DEVD-fmk, a caspase-3-specific inhibitor, blocked SVB-induced apoptosis and increased the survival rate of SVB-treated cells, indicating that activation of caspase-3 plays a key role in SVB-induced apoptosis. In addition, within concentrations that were not cytotoxic to A549 cells, SVB induced marked inhibition of cell motility and invasiveness. Activities of matrix metalloproteinase (MMP)-2 and MMP-9 in AGS cells were dose-dependently inhibited by treatment with SVB, and this was also correlated with a decrease in expression of their mRNA and proteins, and upregulation of tissue inhibitors of metalloproteinase (TIMP)-1 and TIMP-2 mRNA expression. Further studies are needed; however, the results indicated that SVB induces apoptosis of A549 cells through a signaling cascade of death receptor-mediated extrinsic as well as mitochondria-mediated intrinsic caspase pathways. Our data also demonstrated that MMPs are critical targets of SVB-induced anti-invasiveness in A549 cells.

## Introduction

Apoptosis is the active process of endogenous programmed cell death, which plays an important role in developmental processes, maintenance of homeostasis, and elimination of damaged cells. Apoptosis is a tightly regulated process characterized by a series of distinct morphological and biochemical alterations to cells, including plasma membrane blebbing, cell shrinkage, cell surface expression of phosphatidylserine, depolarization of mitochondria, chromatin condensation, and DNA fragmentation. Many gene products have been demonstrated as critical in regulation of apoptosis. In general, apoptosis can be initiated through two well-recognized pathways in cells; the death receptor-mediated (extrinsic) pathway and the mitochondria-dependent (intrinsic) pathway (1,2). In the former case, plasma membrane death receptors are involved and the apoptosis signal is provided by ligation between ligands and cell surface death receptors, and activation of caspase-8, which activates downstream effector caspases (−3, −6, and/or −7). The mitochondrion-mediated intrinsic pathway begins with disruption of mitochondrial membrane potential (MMP, *ΔΨ_m_*) and release of apoptogenic proteins, such as cytochrome *c*, into the cytosol. Once in the cytosol, cytochrome *c* can activate caspase-9, which in turn cleaves and activates caspase-3. Thus, caspases, a group of cysteine proteases, play key roles in both apoptotic pathways. Caspases are synthesized as proenzymes, which are activated by cleavage of the prodomain at a specific aspartic acid cleaving site. Caspase activation is often regulated by various cellular factors, including members of the Bcl-2 family and/or inhibitor of apoptosis (IAP) family proteins (3,4). Although these pathways act independently to initiate apoptosis, a delicate balance and cross-talk between the extrinsic and intrinsic pathways occurs in many cell types. However, most cancer cells block apoptosis, which allows for survival of malignant cells despite genetic and morphologic transformations. Thus, induction of apoptosis in tumor cells has been shown to be the most common anti-cancer mechanism targeted by many cancer therapies (5,6). Therefore, there is a need to identify potential therapeutic anti-tumor agents with potent and cancer cell selective apoptotic effects.

Metastasis is a sequential multi-step process, which ultimately leads to outgrowth of the cancer in a different organ from which it had originated. Metastasis is a major barrier to treatment of cancer and a single event that results in the death of most patients with cancer. This process involves the following steps: invasion of adjacent tissues, intravasation, transport of cancer cells through the circulatory system, arrest at a secondary site, and extravasation and growth in a secondary organ (7–9). Therefore, inhibition of tumor cell migration and invasion are important mechanisms in the anti-metastatic properties of anti-cancer drugs. Recently, substantial data have indicated that inverted expression of matrix metalloproteinases (MMPs) and tissue inhibitors of metalloproteinases (TIMPs) suggest that they function as key regulators in cancer progression, invasion, and metastasis. MMPs, a family of zinc-dependent endopeptidases, are known to process a broad spectrum of cell surface molecules and to function in several important biological processes. They are collectively capable of cleavage of virtually all extracellular matrix (ECM) substrates, and degradation of matrix is a key event in progression, invasion, and metastasis of potentially malignant and malignant lesions (10,11). Among various MMPs, MMP-2 and MMP-9 appear to play an important role in tumor invasion and metastasis and are highly expressed in epithelial cancer cells, including lung carcinoma cells (12–14). On the other hand, TIMPs are naturally occurring inhibitors of MMPs, which inhibit catalytic activity of MMPs through binding to activated MMPs and control of breakdown of ECM (14,15). TIMPs can also inhibit proliferation, invasion, and metastasis of malignant cells. Disturbance in balance of MMPs and TIMPs is found in various pathologic conditions, including cancer (16). Therefore, balance between MMPs and TIMPs plays a vital role in maintaining the integrity of healthy tissues, and MMP inhibitors, as well as TIMP activators, are expected to be useful chemo-therapeutic agents for treatment of malignant cancer.

Amphibian skin extract has been used as a traditional Chinese medicine for centuries for alleviation of human suffering (17). Toads, particularly members of the genus Bufo, have been identified as a particularly useful and readily available source of granular gland secretions, which commonly contain biogenic amines, bufodienolides, alkaloids and steroids, and peptides and proteins (18,19). Chan Su, also called toad venom, is dried white venom prepared from skin secretions of giant toads, such as *Bufo bufo gargarizans* Cantor and *B. melsanostictus* Schneider. It is widely used in clinics for treatment of various cancers and heart failure (20–22). Chan Su has been applied in numerous clinical situations; however, the bioactive compounds in Chan Su are not fully known. Until recently, only a few studies have reported on its pharmacological effects, and bufadienolides, including bufalin, are generally agreed upon to be the major bioactive components (19,23). Several studies have demonstrated the potency of Chan Su and its major components in treatment of various cultured human cancer cells by induction of cell cycle arrest and apoptosis (23–28). The significance of Chan Su in induction of apoptosis against human bladder carcinoma cells, which was mediated by modulation of Bcl-2 family proteins and proteolytic activation of caspases, has recently been reported (29). The apoptotic effects of Chan Su were also associated with specific inhibition of cyclooxygenase-2 expression and prostaglandin E_2_ production (29). The effects of extract of Chan Su induced apoptosis in non-small cell lung cancer (NSCLC) A549 cells accompanied by modulation of the death receptor system, Bcl-2 family members, mitochondrial dysfunction, and activation of caspases have been demonstrated (30). However, the precise anti-cancer effects of Chan Su in human malignant cells are largely unknown.

The present study attempts to elucidate the anti-cancer potential of the whole skin of *Venenum bufonis* (SVB) in the NSCLC A549 cell line and the underlying intracellular signal transduction pathways involved in regulation of apoptosis and metastasis. Results of this study demonstrated that SVB induces apoptosis of A549 cells through a signaling cascade of death receptor-mediated extrinsic and mitochondria-mediated intrinsic caspase pathways. Our data also indicated that SVB inhibits cell motility and invasion of A549 cells through inhibition of the activities of MMPs, while concurrently inducing TIMP expression.

## Materials and methods

### Cell culture and SVB preparation

The NSCLC A549 cell line was obtained from the American Type Culture Collection (Rockville, MD, USA) and cultured in RPMI-1640 medium (Gibco BRL, Gaithersburg, MD, USA) supplemented with 10% heat-inactivated fetal bovine serum (FBS, Gibco BRL), 2 mM glutamine, 100 U/ml penicillin, and 100 μg/ml streptomycin in a humidified environment with 5% CO_2_ at 37°C. The whole skin of *V. bufonis* (SVB) was obtained from Dunsan Oriental Hospital (Daejeon, South Korea). For preparation of extracts of SVB, SVB was pan fried at 90°C for 1 min; 45.4 g of SVB was then washed with distilled water, and boiled in 1 l water at 80°C for 6 h. Solid particles and aggregates were removed by centrifugation at 3,000 × g for 30 min, followed by lyophilisis of the supernatants. Finally, 19.8 g lyophilized SVB were obtained and used in this experiment. The lyophilized extract was stored at −20°C until used or dissolved to a 100 mg/ml concentration with medium, and the stock solution was then diluted with medium to the desired concentration prior to use.

### Cell proliferation and viability assay, and morphological study

For the cell proliferation study, cells were cultured in the absence and presence of variable concentrations of SVB for 24 h. Cells were trypsinized, washed with phosphate-buffered saline (PBS), and viable cells were scored using a hemo-cytometer through exclusion of trypan blue. Measurement of cell viability was determined using the 3-(4,5-dimethylthiazol-2-yl)-2,5-diphenyl-tetrazolium bromide (MTT, Sigma Chemical Co., St. Louis, MO, USA) assay, which is based on the conversion of MTT to MTT-formazan by mitochondrial enzymes. For morphological study, the cells were treated with SVB for 24 h and directly photographed with an inverted microscope (Carl Zeiss, Germany).

### Nuclear staining with DAPI

For evidence of apoptosis, morphological changes of nuclei were visualized following DNA staining using the fluorescent dye 4,6-diamidino-2-phenylindole (DAPI, Sigma). After treatment of A549 cells with SVB, the cells were harvested, washed in ice-cold PBS, and fixed with 3.7% paraformaldehyde (Sigma) in PBS for 10 min at room temperature. Fixed cells were collected using cytospin, washed with PBS, and stained with DAPI solution for 10 min at room temperature. The cells were washed two more times with PBS and the nuclear morphology of the cells was examined using a fluorescence microscope (Carl Zeiss, Germany).

### Flow cytometry analysis for measurement of sub-G1 phase

After treatment with various concentrations of SVB for 24 h, the cells were harvested and washed twice with ice-cold PBS, fixed in ice-cold 70% ethanol, and stored at 4°C. Prior to analysis, the cells were washed once again with PBS, suspended in 1 ml of a cold propidium iodide (PI, Sigma) solution containing 100 μg/ml RNase A, 50 μg/ml PI, 0.1% (w/v) sodium citrate, and 0.1% (v/v) NP-40, and further incubated on ice for 30 min in the dark. DNA content at sub-G1 phase was then determined by flow cytometer (FACSCaliber, Becton-Dikinson, San Jose, CA, USA) and CellQuest software was used for determination of the relative DNA content based on the presence of a red fluorescence (31).

### Mitochondrial membrane potential (MMP, ΔΨ_m_) assay

MMP (*ΔΨ_m_*) values were measured using a flow cytometer with a lipophilic cationic probe 5,5′,6,6′-tetrachloro-1,1′,3,3′-tetra-ethylbenzimidazolylcarbocyanine lodide (JC-1, Calbiochem, San Diego, CA, USA). JC-1 is a ratiometric, dual-emission fluorescent dye that is internalized and concentrated by respiring mitochondria and can reflect changes in MMP (*ΔΨ_m_*) in living cells. There are two excitation wavelengths, 527 nm (green) for the monomer form and 590 nm (red) for the J-aggregate form. With normal mitochondrial function, MMP (*ΔΨ_m_*) is high and the red fluorescence is predominant. However, when there is mitochondrial injury, MMP (*ΔΨ_m_*) is reduced, leading to an increase in green fluorescence. Quantitation of red and green fluorescent signals reflects whether mitochondria are damaged. For this study, cells treated with SVB were trypsinized and the cell pellets were re-suspended in 500 μl of PBS and incubated with 10 μM JC-1 for 20 min at 37°C. The cells were subsequently washed once with cold PBS, suspended in a total volume of 500 μl, and analyzed using a flow cytometer.

### Protein extraction, gel electrophoresis, and Western blot analysis

Cells were treated with SVB for 24 h and harvested with ice-cold PBS. Total cell lysates were lysed in an extraction buffer [25 mM Tris-Cl (pH 7.5), 250 mM NaCl, 5 mM ethylenediaminetetra acetic acid, 1% Nonidet P-40, 0.1 mM sodium orthovanadate, 2 μg/ml leupeptin, and 100 μg/ml phenylmethylsulfonyl fluoride]. A Bio-Rad protein assay kit (Bio-Rad, Hercules, CA, USA) was used for determination of protein concentration. For Western blot analysis, proteins (~30–50 μg) were separated by ~8–10% sodium dodecyl sulfate (SDS)-polyacrylamide gel electrophoresis and then electrotransferred to a nitrocellulose membrane (Schleicher & Schuell, Keene, NH, USA). Membranes were blocked with 5% skim milk for 1 h and then subjected to immunoblot analysis with the appropriate antibodies. Proteins were then visualized by the enhanced chemiluminescence (ECL) method according to the recommended procedure (Amersham Co., Arlington Heights, IL, USA). Primary antibodies were purchased from Santa Cruz Biotechnology Inc. (Santa Cruz, CA, USA) and Calbiochem. Peroxidase-labeled donkey anti-rabbit immunoglobulin and peroxidase-labeled sheep anti-mouse immunoglobulin were purchased from Amersham (32).

### Assay of caspase-3, -8 and -9 activity

Enzymatic activity of caspases induced by SVB was assayed using a colorimetric assay kit according to the manufacturer’s protocol (R&D Systems, Minneapolis, MN, USA). Briefly, the cells were lysed in a lysis buffer for 30 min on an ice bath. The lysed cells were centrifuged at 12,000 g for 10 min, and 100 μg of the protein was incubated with 50 μl of a reaction buffer and 5 μl of the colorimetric tetrapeptides, Asp-Glu-Val-Asp (DEVD)-p-nitroaniline (pNA) for caspase-3, Ile-Glu-Thr-Asp (IETD)-pNA for caspase-8 and Leu-Glu-His-Asp (LEHD)-pNA for caspase-9, respectively, at 37°C for 2 h. Optical density of the reaction mixture was quantified spectrophotometrically at a wavelength of 405 nm (33).

### Wound healing migration assay

Wound healing experiments were conducted in order to assess the effect of SVB on A549 cell motility. In brief, cells were grown to confluence on 30-mm cell culture dishes coated with rat tail collagen (20 μg/ml, BD Biosciences, Bedford, MA, USA), and then treated for 6 h with vehicle or 2 μg/ml of SVB, which induced no cytotoxic effects, as shown by the results of the MTT assay. A scratch was made in the cell layer using a pipette tip. After washing with PBS, serum-free media (to prevent cell proliferation) containing either vehicle or SVB was added. In order to monitor cell movement into the wounded area, photographs of the wounded area were taken immediately after the scratch was made and 12 and 24 h later.

### Matrigel invasion assay

In order to determine the effects of SVB on A549 cell invasiveness, the cells were exposed for 6 h to 2 μg/ml of SVB, and were evaluated by the Boyden chamber (BD Biosciences) invasion assay. Briefly, treated cells (50,000) were plated onto the apical side of Matrigel-coated filters in serum-free medium containing either vehicle or SVB. Medium containing 20% FBS was placed in a basolateral chamber as a chemoattractant. After 24 h, cells on the apical side were wiped off with a Q-tip. Cells on the bottom of the filter were stained with hematoxylin (Sigma) and counted (three fields of each triplicate filter) using an inverted microscope.

### RNA extraction and reverse transcription-PCR

Total RNA was prepared using an RNeasy kit (Qiagen, La Jolla, CA, USA) and primed with random hexamers for synthesis of complementary DNA using AMV reverse transcriptase (Amersham Corp.) according to the manufacturer’s instructions. Polymerase chain reaction (PCR) was performed in a Mastercycler (Eppendorf, Hamburg, Germany) with the primers indicated in Table I. Conditions for PCR reactions were 1× (94°C for 3 min), 35× (94°C for 45 sec; 58°C for 45 sec; and 72°C for 1 min) and 1× (72°C for 10 min). Amplification products obtained by PCR were electrophoretically separated on 1% agarose gel and visualized by ethidium bromide (EtBr) staining.

### Gelatin zymographic analysis of secreted MMPs

After incubation with SVB for 24 h, cell culture supernatants were collected and centrifuged at 400 × g for 5 min. The cell-free supernatant was mixed with 2X sample buffer (Invitrogen) and zymography was performed using precast gels (10% polyacrylamide and 0.1% gelatin). Following electrophoresis, the gels were washed twice at room temperature for 30 min in 2.5% Triton X-100, subsequently washed in buffer containing 50 mM Tris-HCl, 150 mM NaCl, 5 mM CaCl_2_, 1 μM ZnCl_2_, 0.02% NaN_3_ at pH 7.5 and incubated in this buffer at 37°C for 24 h. Thereafter, the gels were stained with 0.5% (w/v) Coomassie brilliant blue G-250 (Bio-Rad) for 1 h, then lightly de-stained in methanol:acetic acid:water (3:1:6). Clear bands appear on the Coomassie stained blue background in areas of gelatinolytic activity. Gels were scanned and images were processed by extraction of the blue channel signal, converting it to black and white and inverting it in order to quantify the gelatinolytic activities from the integrated optical density.

### Statistical analysis

Data are expressed as the means ± SD. Statistical comparisons were performed using One-way ANOVA followed by a Fisher’s test. Significant differences between the groups were determined using an unpaired Student’s t-test. A p<0.05 was considered significant.

## Results

### Growth inhibition and apoptosis induction by SVB in A549 cells

**Figure 1:**
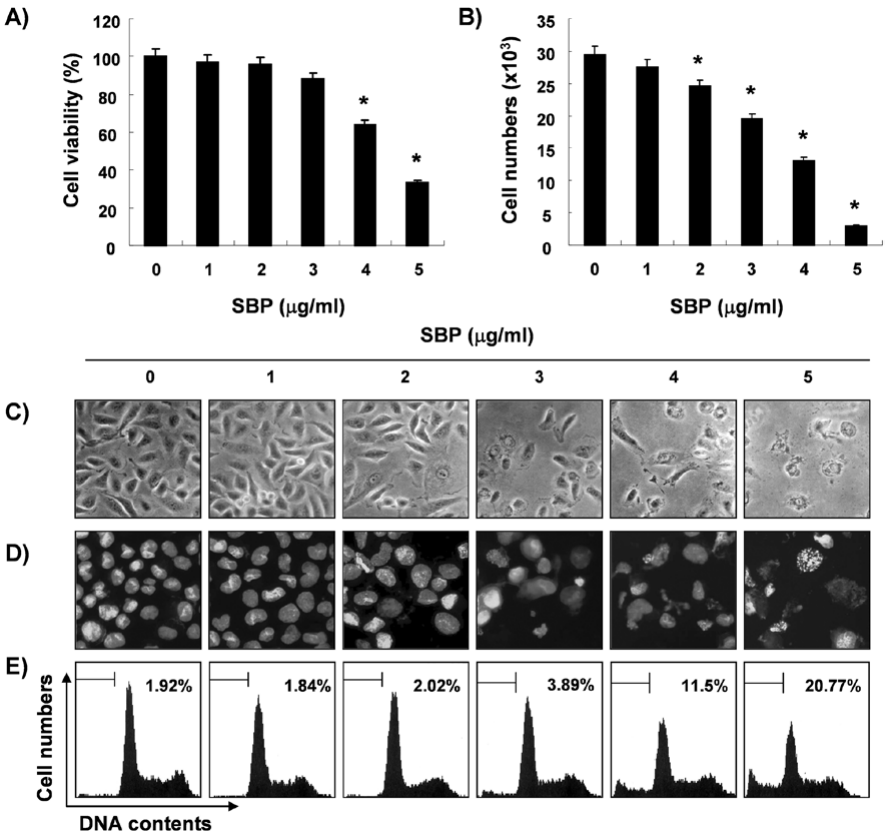
Inhibition of cell growth and induction of apoptosis by SVB treatment in A549 human lung carcinoma cells. Cells were plated at 4×10^4^ cells per 60-mm plate, and incubated for 24 h. Cells were treated with varying concentrations of SVB for 24 h, and cell viability and proliferation were measured by the metabolic-dye-based MTT assay (A) and hemocytometer counts of trypan blue-excluding cells (B), respectively. Data are expressed as mean ± SD of three independent experiments. Values marked as ^*^ indicate significant differences from other treatments (^*^p<0.05). (C) Following treatment of cells with the indicated concentrations of SVB, they were observed using an inverted microscope (magnification ×200). (B) Cells grown under the same conditions as (C) were stained with DAPI for 10 min, washed with PBS, and then photographed with a fluorescence microscope using a blue filter (magnification ×400). (E) To quantify the degree of apoptosis induced by SVB, cells grown under the same conditions were evaluated for sub-G1 DNA content, which represents fractions undergoing apoptotic DNA degradation, using a flow cytometer. Each point represents the average of two independent experiments.

In order to determine whether SVB inhibits cell viability and proliferation, A549 cells were treated with various concentrations of SVB and the relative cell proliferation and viable cell number were then measured by the MTT assay and trypan blue exclusion method, respectively. As shown in Fig. 1A and B, treatment with SVB resulted in a significant reduction in cell proliferation and viability, and these effects occurred in a concentration-dependent manner. Next, experiments were performed in order to determine whether the inhibitory effects of SVB on cell viability and proliferation are the result of apoptotic cell death. Direct observation using an inverted microscope showed many distinct morphological changes in cells treated with SVB, compared with control cells (Fig. 1C). In particular, cell shrinkage, cytoplasm condensation, and formation of cytoplasmic filaments with protuberances resulted in more of a spindle shape, membrane shrinkage, and cell rounding up appeared in a concentration-dependent manner after SVB treatment. Using morphological analysis with DAPI staining, nuclei with chromatin condensation and formation of apoptotic bodies were observed in cells cultured with SVB in a concentration-dependent manner. In contrast, very few were observed in the control culture (Fig. 1D). We further analyzed the amount of sub-G1 DNA, which contained less DNA than G1 cells, in order to quantify the degree of apoptosis induction of A549 cells by SVB treatment using a flow cytometer. Flow cytometric analysis indicated that SVB treatment caused significant increases in apoptotic cell percentages, as compared with control cells (20% of apoptotic cells in cells with 5 μg/ml of SVB for 24 h, Fig. 1E). These results demonstrated an association of the cytotoxic effects observed in response to SVB with induction of apoptosis in A549 cells, and there was a good correlation between the extent of apoptosis and inhibition of cell viability and proliferation.

### Effects of SVB on the mitochondrial pathway

**Figure 2:**
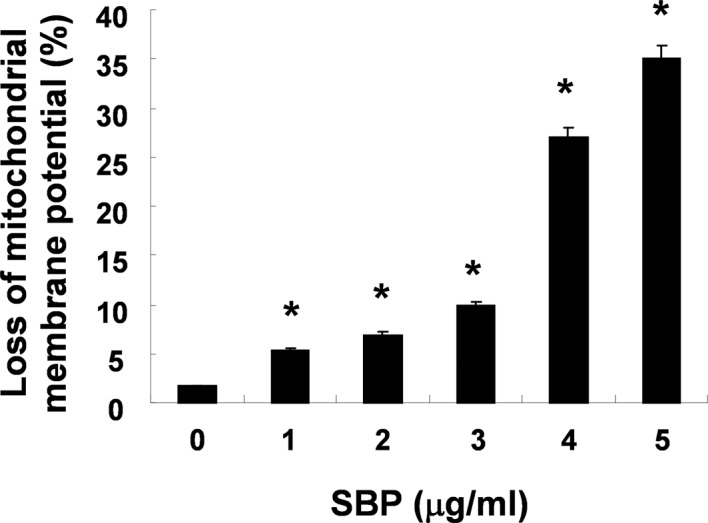
Loss of MMP (*ΔΨ_m_*) by SVB treatment in A549 cells. Cells grown under the same conditions as those in Fig. 1 were stained with JC-1 and then incubated at 37°C for 20 min, after which the mean JC-1 fluorescence intensity was detected using a flow cytometer. Data represent the mean ± SD of representative experiments performed at least three times. Significance was determined by a Student’s t-test (^*^p<0.05 vs. untreated control).

**Figure 3:**
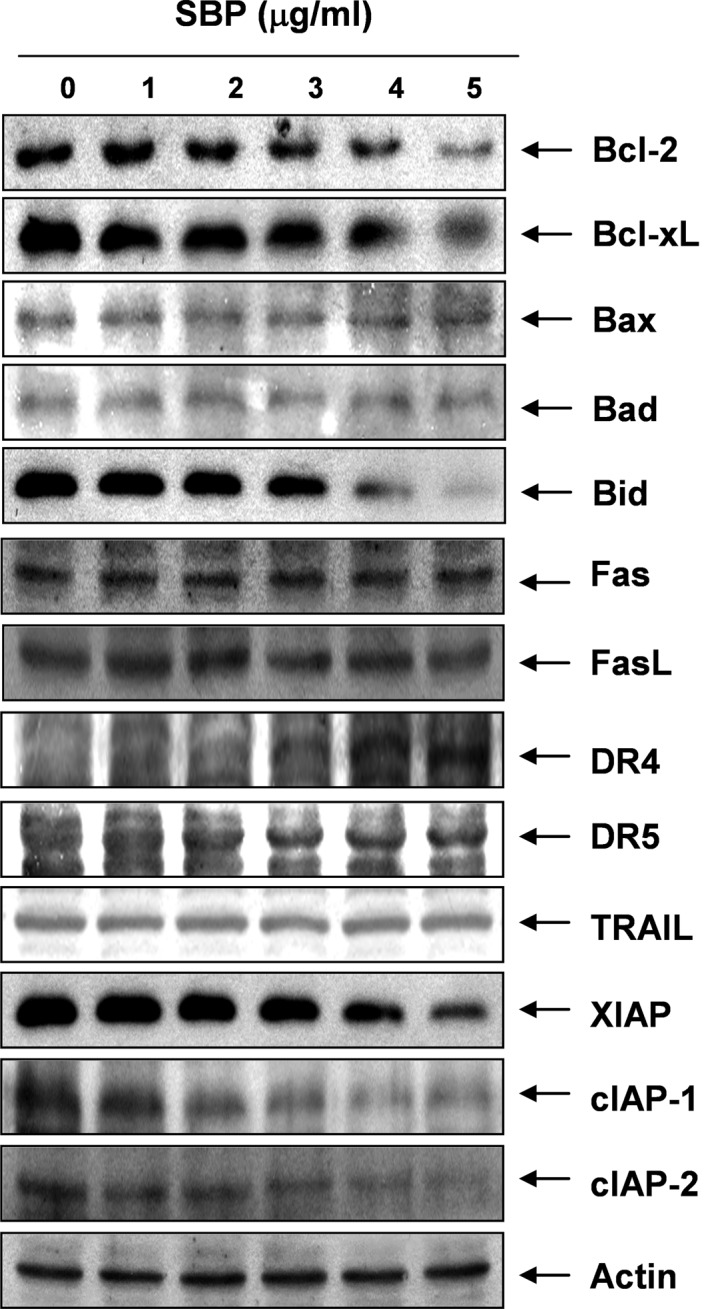
Effects of SVB treatment on levels of apoptosis-related proteins in AGS cells. Cells were treated with the indicated concentrations of SVB for 24 h. Equal amounts of cell lysates were resolved on SDS-polyacrylamide gels and transferred to nitrocellulose membranes. Membranes were probed with the indicated antibodies, and proteins were visualized using the ECL detection system. Actin was used as an internal control.

To investigate the association with the mitochondria-mediated intrinsic pathway in SVB-induced apoptosis, alterations in MMP (*ΔΨ_m_*) were determined using the fluorescent dye, JC-1. As shown in Fig. 2, treatment with SVB resulted in markedly induced mitochondrial membrane hyperpolarization in a concentration-dependent manner. We next examined the expression levels of Bcl-2 family proteins directly interacting with mitochondria. Western blot analyses data revealed that the levels of Bax and Bad expression, pro-apoptotic proteins, remained virtually unchanged in response to SVB treatment, whereas the levels of Bcl-2 and Bcl-xL, anti-apoptotic proteins, were significantly down-regulated by SVB treatment, suggesting that SVB alters the Bax (Bad):Bcl-2 and Bax (Bad):Bcl-XL ratio in A549 cells in a concentration-dependent fashion (Fig. 3). These results suggested that SVB induced apoptotic cell death in A549 cells through the intrinsic mitochondrial pathway, as evidenced by an increase in the ratio of Bax (Bad)/Bcl-2 (Bcl-xL) expression and mitochondrial dysfunction. In addition, although the truncated form of pro-apoptotic protein Bid, a BH3-only protein member of the Bcl-2 family, was not detected, SVB treatment resulted in a decrease of the whole form of Bid proteins. These data indicate the possibility that both the extrinsic and intrinsic pathways might be involved in SVB-induced apoptosis in A549 cells.

### Activation of caspases and degradation of PARP and β-catenin proteins by SVB treatment

**Figure 4:**
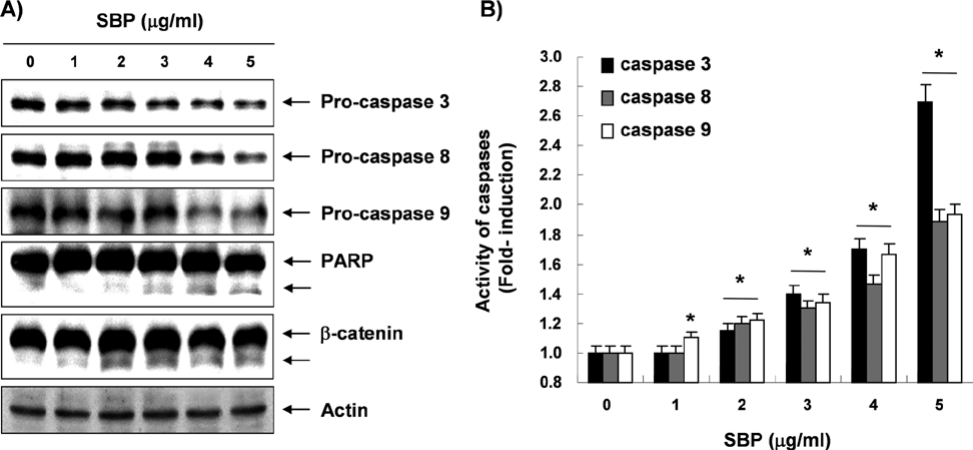
Activation of caspases and degradation of PARP and β-catenin proteins by SVB treatment in AGS cells. (A) Cells treated with various concentrations of SVB for 24 were lysed and cellular proteins were separated by SDS-polyacrylamide gels and transferred onto nitrocellulose membranes. Membranes were probed with anti-caspase-3, -8, and -9, anti-PARP, and anti-β-catenin antibodies. Proteins were visualized using the ECL detection system. Actin was used as an internal control. (B) Cells grown under the same conditions as (A) were collected and lysed. Aliquots were incubated individually with DEVD-pNA, IETD-pNA, and LEHD-pNA for caspase-3, -8, and -9 at 37°C for 1 h. Released fluorescent products were measured. Data represent the mean of three independent experiments. The statistical significance of results was analyzed by a Student’s t-test (^*^p<0.05).

Next, experiments were performed in order to characterize the role of caspase activation in SVB-mediated apoptosis in A549 cells. Immunoblotting results showed markedly decreased levels of pro-caspase-3, -8, and -9 proteins in a concentration-dependent manner in SVB-treated A549 cells (Fig. 4A). Furthermore, in order to monitor the enzymatic activity of these enzymes during SVB-induced apoptosis, *in vitro* caspase activity was measured following treatment with SVB using specific fluorogenic peptide substrates for each caspase. As shown in Fig. 4B, activities of these caspases were significantly increased in a concentration-dependent fashion, as compared with untreated control cells. Subsequent Western blot analyses showed progressive proteolytic cleavage of poly(ADP ribose) polymerase (PARP) and β-catenin proteins, and accumulation of their cleavage forms, which are substrate proteins of caspase-3 (34,35) in A549 cells after SVB treatment (Fig. 4A). The data suggested that activation of caspases is clearly involved in the SVB-induced apoptotic pathway.

### Effects of SVB on levels of the death-receptor pathway and IAP family proteins

In order to determine whether the extrinsic apoptotic pathway was involved in SVB-induced apoptosis, we used Western blot analyses for measurement of expression of death receptors and corresponding pro-apoptotic ligands. As shown in Fig. 3, no significant changes of Fas, Fas ligand (FasL), necrosis factor-related apoptosis-inducing ligand (TRAIL), and death receptor (DR) 5 protein levels were noted in A549 cells treated with SVB; however, SVB induced markedly increased expression levels of DR4 proteins in a concentration-dependent manner, suggesting that the extrinsic apoptotic pathway is also engaged in SVB-induced apoptotic cell death. Furthermore, expression levels in SVB-treated A549 cells were also examined in order to determine whether SVB induces A549 cell death through a change in expression of IAP family proteins, which binds caspases and leads to caspase inactivation for an anti-apoptotic effect (36). As shown in Fig. 3, all of the IAP family proteins examined in this study, including X-linked inhibitor of apoptosis protein (XIAP), cellular inhibitor-of-apoptosis protein (cIAP)-1, and cIAP-2, were concentration-dependently down-regulated in A549 cells treated with SVB.

### Inhibition of SVB-induced apoptosis by caspase-3 inhibitor

**Figure 5:**
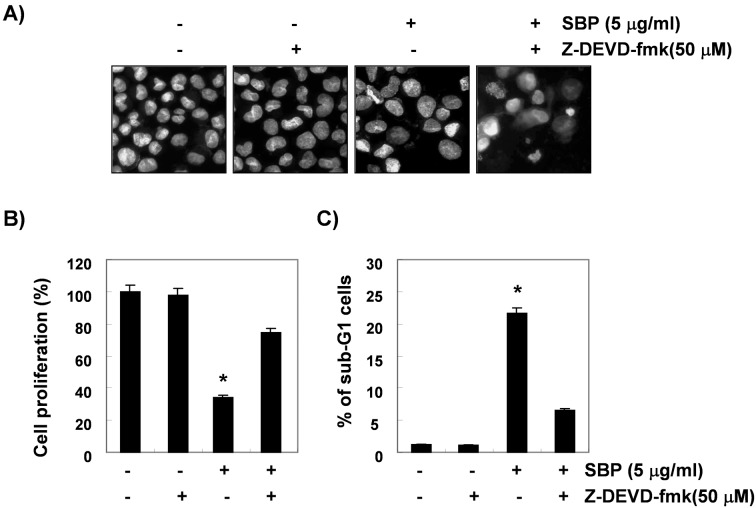
Inhibition of SVB-induced apoptosis by caspase-3 inhibitor in A549 cells. (A) A549 cells were treated with z-DEVD-fmk (50 μM) for 1 h before challenge with 5 μg/ml of SVB for 24 h. Cells were stained with DAPI for 10 min and photographed with a fluorescence microscope using a blue filter (magnification ×400). (B) Cell proliferation was determined using the MTT assay after 24 h in the presence of the caspase-3 inhibitor z-DEVD-fmk (50 μM) for 1 h before SVB (5 μg/ml) treatment. Data are expressed as mean ± SD of three independent experiments. (C) Cells grown under the same conditions as (A) were evaluated for sub-G1 DNA content using a flow cytometer. Data are reported as mean ± SD of three independent experiments. The significance was determined by Student’s t-test (^*^p<0.05 vs. untreated control).

To show that activation of caspase-3 is a key step in the SVB-induced apoptotic pathway, A549 cells were pretreated with z-DEVD-fmk (50 μM), a cell-permeable caspase-3 inhibitor, for 1 h, followed by treatment with 5 μg/ml of SVB for 24 h. Blockade of caspase-3 activity by pretreatment of cells with z-DEVD-fmk prevented SVB-induced chromatin condensation, growth inhibition, and the increase in the sub-G1 population (Fig. 5). These results clearly demonstrated an association of SVB-induced apoptosis with activation of caspase-3 and that activation of caspase-3 plays an important role in SVB-induced apoptosis in A549 cells.

### Inhibition of cell motility and cell invasion by SVB in A549 cells

**Figure 6:**
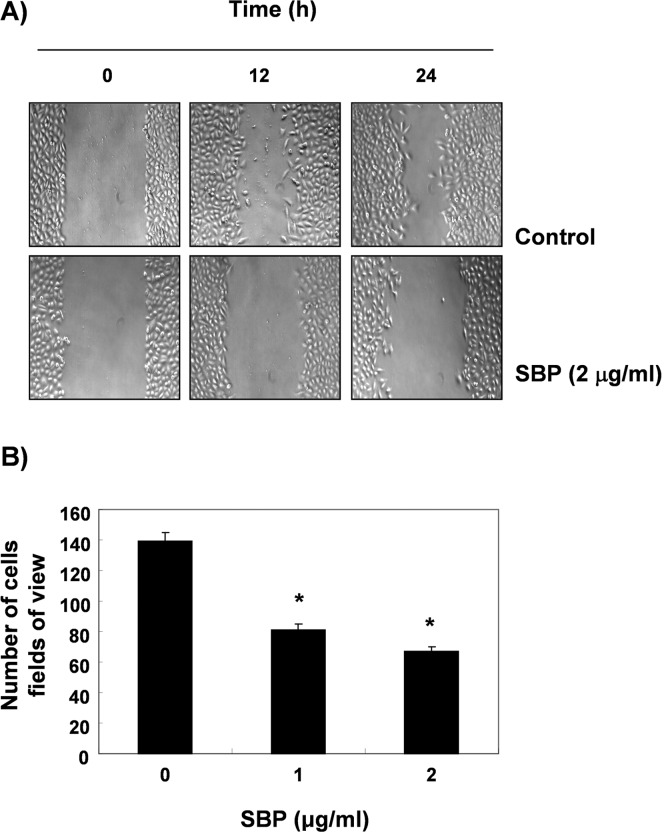
Inhibition of cell motility and invasion by SVB treatment in A549 cells. (A) Cells were grown to confluency on 30-mm cell culture dishes and then a scratch was made through the cell layer using a pipette tip. After washing with PBS, serum-free media (to prevent cell proliferation) containing either vehicle or 2 μg/ml of SVB was added for 48 h. Photographs of the wounded area were taken in order to evaluate cell movement into the wounded area. (B) Cells pretreated with the indicated concentrations of SVB for 6 h were plated onto the apical side of matrigel coated filters in serum-free medium containing either vehicle or SVB. Medium containing 20% FBS was placed in the basolateral chamber to act as a chemoattractant. After 48 h, cells on the apical side were wiped off using a Q-tip. Next, cells on the bottom of the filter were stained using hematoxylin and eosin Y, and were then counted. Data are shown as the mean of triplicate samples and represent invasive cell numbers, compared with those of control cells. The significance was determined using a Student’s t-test (^*^p<0.05 versus untreated control).

A wound healing experiment was performed. In order to determine whether SVB inhibits the cell motility of A549 cells. The results, as shown in Fig. 6A, demonstrated that 2 μg/ml of SVB, which was not cytotoxic, as shown by the MTT assay, resulted in time-dependent delay of the cell motility of A549 cells, as compared with control cells. Using a Boyden chamber invasion assay, we next examined the question of whether or not SVB decreases the activity of cell invasion. As shown in Fig. 6B, SVB treatment resulted in reduced cell invasion through the Matrigel chamber in a concentration-dependent manner, suggesting that inhibition of cell motility by SVB was associated with inhibition of cell invasion in A549 cells.

### Down-regulated activities and expression of MMPs by SVB in A549 cells

**Figure 7:**
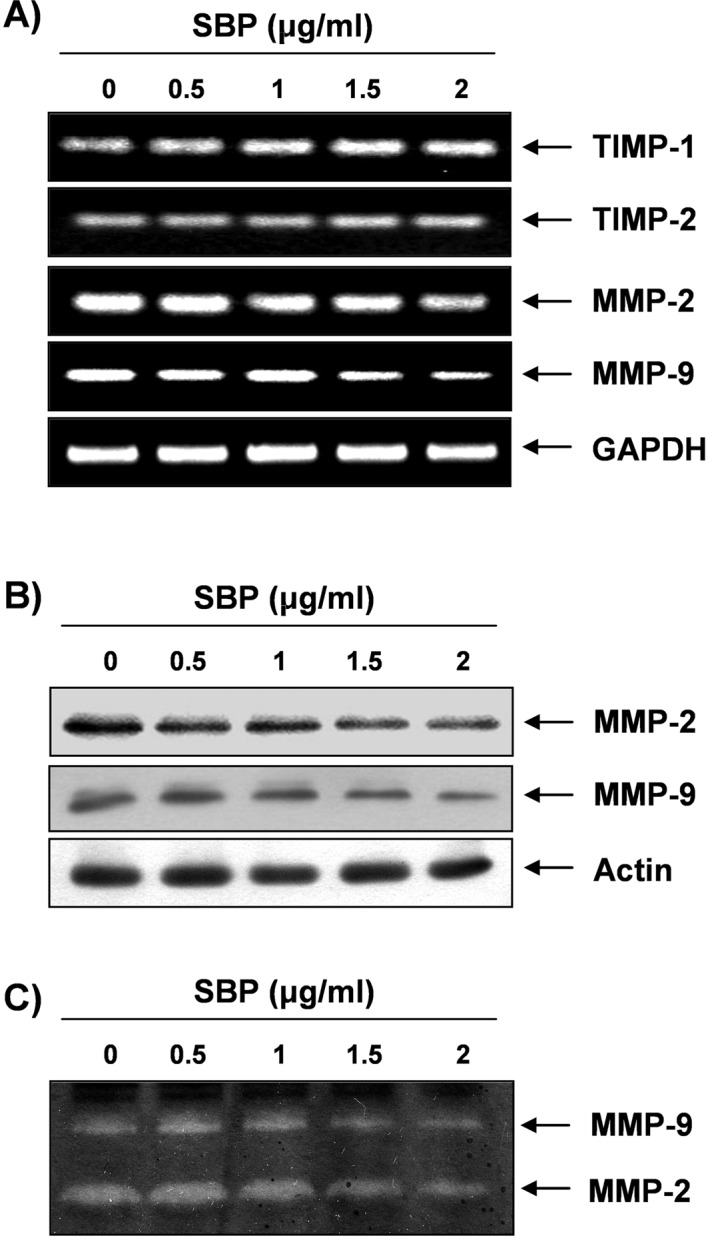
Effect of SVB treatment on expression of TIMPs and MMPs, and activity of MMPs in A549 cells. (A) Cells were treated with the indicated concentrations of SVB for 24 h. Total RNAs were isolated and reverse-transcribed. The resulting cDNAs were then subjected to PCR with TIMP-1, TIMP-2, MMP-2, and MMP-9 primers and the reaction products were subjected to electrophoresis in a 1% agarose gel and visualized by EtBr staining. Representative results from two independent experiments are shown. GAPDH was used as an internal control. (B) Cells grown under the same conditions as (A) were lysed and cellular proteins were separated by electrophoresis on SDS-polyacrylamide gels. Western blotting was then performed using anti-MMP-2 and anti-MMP-9 antibodies, and an ECL detection system. Actin was used as an internal control. (C) Following incubation with the indicated concentrations of SVB for 24 h, medium was collected, and the activities of MMP-2 and MMP-9 were measured by zymo-graphy.

Cell migration plays an important role in the process of metastasis, and invasion of the basement membrane is primarily mediated by gelatinase MMPs (10,11); therefore, we tested the effects of SVB on TIMP and MMP mRNA levels by RT-PCR. As shown in Fig. 7A, SVB treatment resulted in slightly increased TIMP-1 and -2 mRNA levels; however, MMP-2 and -9 levels were decreased in a concentration-dependent manner. We next investigated the effects of SVB on protein levels and activities of MMPs using Western blot analysis and gelatin zymography. Data indicated that activities of MMP-2 and -9 in A549 cells were decreased by SVB treatment, which was connected with a concurrent down-regulation of their mRNA and protein levels and up-regulation of the levels of TIMP-1 and -2 (Fig. 7B and C). These results suggest an association of the anti-invasive effect of SVB with increased TIMP-1 and -2 levels, as well as inhibition of MMP-2 and -9 mRNA, protein expression, and activity in A549 cells.

## Discussion

Recent studies have reported that the extracts of Chan Su or its components can cause cell cycle arrest and apoptosis in various human cancer cell lines, which suggests that their growth inhibitory effect occurred through blockade of the G1/S or G2/M phase, and that these cancer cells do not enter cell cycle progression and die through apoptosis (24–30,37–40). However, the mechanisms responsible for the apoptotic and anti-invasive effects of the whole skin of V. bufonis (SVB) have yet to be determined. Therefore, the aim of this study was to determine the capacity of SVB to induce apoptosis and inhibit invasion, and to identify the biochemical mechanisms in the NSCLC A549 cell line. Our present results demonstrated that SVB significantly inhibits A549 cell growth by induction of apoptotic cell death through modulation of several apoptosis related proteins and activation of caspase. In addition, SVB exhibited anti-invasive activity of A549 cells through inhibition of MMPs activity.

Apoptosis can be triggered by various stimuli, including death receptor-mediated signaling (the death receptor/extrinsic pathway) and intracellular stress (the mitochondrial/intrinsic pathway). In addition to the energy source, mitochondria are known as major regulators of extrinsic as well as intrinsic apoptosis pathways, and they undergo a series of consequential changes during apoptosis. Mitochondrial function is controlled by several factors, such as the Bcl-2 and IAP family proteins, and activity of caspases (1,2,6). Members of the Bcl-2 family are significantly involved in regulation of apoptosis, either as an activator (e.g., Bax, and Bad) or as an inhibitor (e.g., Bcl-2, and Bcl-xL); therefore, it has been suggested that the Bax/Bcl-2 ratio is a key factor in regulation of the apoptotic process (41,42). Members of the IAP family function through binding to and inhibition of several caspases (43,44). Activation of the intrinsic/mitochondrial apoptosis pathway leads to disruption of MMP (*ΔΨ_m_*) and release of apoptogenic proteins, such as cytochrome *c*, which removes IAP blockage of caspase activation (45,46). The present data showed that SVB-induced apoptosis was related to down-regulation of pro-apoptotic Bcl-2 and Bcl-xL proteins without alteration of pro-apoptotic Bax and Bad expression (Fig. 3). Furthermore, exposure of A549 cells to SVB resulted in a loss of MMP (*ΔΨ_m_*) (Fig. 2) and down-regulation of IAP family proteins, including XIAP, cIAP-1 and cIAP-2 (Fig. 3). The data suggest that SVB induced an increase in the Bax (or Bad)/Bcl-2 (or Bcl-xL) ratio and induced mitochondrial dysfunction, leading to apoptosis in A549 cells.

Cell surface death ligand/receptor systems, such as Fas/FasL and TRAIL/DRs, are key signaling transduction pathways of the extrinsic pathway of apoptosis in cells. Binding FasL to Fas receptors and/or TRAIL to DRs leads to receptor oligomerization and formation of the death-inducing signaling complex, followed by activation of caspase-8, and then cleavage of Bid (tBid). tBid can translocate to mitochondria and bind to Bax, leading to a conformational change of Bax and to activation of caspase-9, and concomitant activation of caspase-3 (45,47). Thus, caspase-3 is the most important executioner of apoptosis. Significant evidence has indicated that caspase-3 is either partially or totally responsible for proteolytic cleavage of many key proteins, including PARP and β-catenin, which are marker proteins for apoptosis (34,35). Thus, the levels of death receptor-related proteins, the catalytic activity of caspases, and the levels of Bid were next examined in order to further gain mechanical insights into SVB-induced apoptosis of A549 cells. The data demonstrated that SVB induced an increase in the levels of DR4, the enzymatic activity of extrinsic and intrinsic caspase cascades, such as caspase-8 and -9, and decreased the levels of total Bid expression (Figs. 3 and 4). In addition, SVB-induced apoptosis was associated with increased activities of caspase-3 in a concentration-dependent fashion and a concomitant degradation of PARP and β-catenin, and cleavage fragments of both proteins showed a gradual increase in SVB-treated A549 cells (Fig. 4). However, under the same conditions, a specific caspase-3 inhibitor, z-DEVD-fmk, was able to prevent SVB-induced apoptosis (Fig. 5) showing that activation of caspase-3 contributed to SVB-induced apoptosis. Thus, the results of this study demonstrate that SVB triggers apoptosis of A549 cells through activation of the intrinsic caspase pathway along with the death receptor-mediated extrinsic pathway.

Metastasis is the process of spread of cancer cells to tissues and organs beyond where the tumor originated and formation of new tumors. The process ultimately leads to outgrowth in a different organ from which it had originated (5,7,8). Cancer cell invasion and migration are critical steps during metastasis; therefore, their inhibition is an important mechanism for anti-cancer drugs. Endopeptidase MMPs play important roles in cancer invasion and metastasis; therefore, tumor metastasis can be inhibited by blockade of MMP synthesis and activity (14,48). Many researchers have reported that the anti-metastatic actions of natural products, including phytochemical agents, were associated with a reduction in MMP-2 and MMP-9 activity (49–53). MMP activity is tightly controlled by transcriptional activation, by a complex proteolytic activation cascade, and by an endogenous system of TIMPs. TIMPs inhibit MMPs by formation of 1:1 stoichiometric complexes for regulation of matrix turnover (15,16). Treatment with <2 μg/ml of SVB, which was not cytotoxic, resulted in markedly inhibited cell motility and invasive activity in AGS cells (Fig. 6); therefore, the question of whether or not the inhibitory effects of SVB were associated with modulation of TIMP and MMP expression or their activities was investigated. Our results indicated that SVB induced marked inhibition of MMP-2 and MMP-9 mRNA and protein levels, as well as their enzymatic activities in a concentration-dependent manner (Fig. 7). However, the transcriptional levels of both TIMP-1 and TIMP-2 showed concentration-dependent up-regulation in response to SVB treatment, demonstrating that SVB-induced inhibition of cell motility and invasion is related to down-regulation of MMP-2 and MMP-9 activities through elevation of TIMP expression. Therefore, the results suggested that SVB may induce an increase in the TIMPs/MMPs ratio as a key factor in regulation of the anti-invasive process, which subsequently blocks degradation of ECM and leads to inhibited cell invasion.

In conclusion, the present results indicate that SVB induces significant suppression of proliferation of A549 cells by induction of apoptosis through activation of the mitochondrial mediated-intrinsic caspase pathway along with the death receptor-mediated extrinsic pathway. The present data also revealed that SVB has an anti-invasive property, which is accompanied by repression of MMPs activities while concurrently inducing TIMPs expression. Although it is still unclear whether SVB can induce apoptosis and inhibit metastasis through other pathways, the results provide new information on possible mechanisms for the anti-cancer activity of SVB; Chan Su is a promising candidate for cancer chemoprevention and/or chemotherapy as well as decreasing the risk of development of cancer.

## Figures and Tables

**Table I:** Sequences of the primer pairs employed in the RT-PCR reactions.

Name		Sequence of primers
TIMP-1	Sense	5′-TGG-GGA-CAC-CAG-AAG-TCA-AC-3′
	Antisense	5′-TTT-TCA-GAG-CCT-TGG-AGG-AG-3′
TIMP-2	Sense	5′-GTC-AGT-GAG-AAG-GAA-GTG-GAC-TCT-3′
	Antisense	5′-ATG-TTC-TTC-TCT-GTG-ACC-CAG-TC-3′
MMP-2	Sense	5′-GGC-CCT-GTC-ACT-CCT-GAG-AT-3′
	Antisense	5′-GGC-ATC-CAG-GTT-ATC-GGG-GA-3′
MMP-9	Sense	5′-CGG-AGC-ACG-GAG-ACG-GGT-AT3′
	Antisense	5′-TGA-AGG-GGA-AGA-CGC-ACA-GC-3′
GAPDH	Sence	5′-CGG-AGT-CAA-CGG-ATT-TGG-TCG-TAT-3′
	Antisense	5′-AGC-CTT-CTC-CAT-GGT-GGT-GAA-GAC-3′

## References

[1] Jin Z, El-Deiry WS (2005). Overview of cell death signaling pathways. Cancer Biol Ther.

[2] Wesche-Soldato DE, Swan RZ, Chung CS, Ayala A (2007). The apoptotic pathway as a therapeutic target in sepsis. Curr Drug Targets.

[3] Earnshaw WC, Martins LM, Kaufmann SH (1999). Mammalian caspases: structure, activation, substrates, and functions during apoptosis. Annu Rev Biochem.

[4] Stennicke HR, Salvesen GS (1998). Properties of the caspases. Biochim Biophys Acta.

[5] Makin G, Dive C (2001). Apoptosis and cancer chemotherapy. Trends Cell Biol.

[6] Ghobrial IM, Witzig TE, Adjei AA (2005). Targeting apoptosis pathways in cancer therapy. CA Cancer J Clin.

[7] Hart IR, Goode NT, Wilson RE (1989). Molecular aspects of the metastatic cascade. Biochem Biophys Acta.

[8] Jiang WG, Puntis MCA, Hallet MB (1994). The molecular and cellular basis of cancer invasion and metastasis and its implications for treatment. Br J Surg.

[9] Martin TA, Jiang WG (2009). Loss of tight junction barrier function and its role in cancer metastasis. Biochim Biophys Acta.

[10] Duffy MI, Maguire TM, Hill A, McDermott E, O’Higgins N (2000). Metalloproteinases: role in breast carcinogenesis, invasion and metastasis. Breast Cancer Res.

[11] Vihinen P, Ala-aho R, Kähäri VM (2005). Matrix metalloproteinases as therapeutic targets in cancer. Curr Cancer Drug Targets.

[12] Gibbs DF, Warner RL, Weiss SJ, Johnson KJ, Varani J (1999). Characterization of matrix metalloproteinases produced by rat alveolar macrophages. Am J Respir Cell Mol Biol.

[13] Cockett MI, Murphy G, Birch ML, O’Connell JP, Crabbe T, Millican AT, Hart IR, Docherty AJ (1998). Matrix metalloproteinases and metastatic cancer. Biochem Soc Symp.

[14] Matrisian LM (1992). The matrix-degrading metalloproteinases. Bioessays.

[15] Uzui H, Harpf A, Liu M, Doherty TM, Shukla A, Chai N (2002). Increased expression of membrane type 3-matrix metalloproteinase in human atherosclerotic plaque: role of activated macrophages and inflammatory cytokines. Circulation.

[16] Lambert E, Dasse E, Haye B, Petitfrere E (2004). TIMPs as multifacial proteins. Crit Rev Oncol Hematol.

[17] Chen KK, Kovarikova A (1967). Pharmacology and toxicology of toad venom. J Pharm Sci.

[18] Clarke BT (1997). The natural history of amphibian skin secretions, their normal functioning and potential medical applications. Biol Rev.

[19] Steyn PS, van Heerden FR (1998). Bufadienolides of plants and animal origin. Nat Prod Rep.

[20] Nogawa T, Kamano Y, Yamashita A, Pettit GR (2001). Isolation and structure of five new cancer cell growth inhibitory bufadienolides from the Chinese traditional drug Ch’an Su. J Nat Prod.

[21] Bhuiyan MB, Fant ME, Dasgupta A (2003). Study on mechanism of action of Chinese medicine Chan Su: dose-dependent biphasic production of nitric oxide in trophoblastic BeWo cells. Clin Chim Acta.

[22] Ye M, Guo DA (2005). Analysis of bufadienolides in the Chinese drug Chan Su by high-performance liquid chromatography with atmospheric pressure chemical ionization tandem mass spectrometry. Rapid Commun Mass Spectrom.

[23] Wang SW, Lin H, Tsai SC (1998). Effects of methanol extract of Chansu on hypothalamic-pituitary-testis function in rats. Metabolism.

[24] Watabe M, Kawazoe N, Masuda Y, Nakajo S, Nakaya K (1997). Bcl-2 protein inhibits bufalin-induced apoptosis through inhibition of mitogen-activated protein kinase activation in human leukemia U937 cells. Cancer Res.

[25] Kawazoe N, Watabe M, Masuda Y, Nakajo S, Nakaya K (1999). Tiam1 is involved in the regulation of bufalin-induced apoptosis in human leukemia cells. Oncogene.

[26] Giri B, Gomes A, Debnath A, Saha A, Biswas AK, Dasgupta SC, Gomes A (2006). Antiproliferative, cytotoxic and apoptogenic activity of Indian toad (*Bufo melanostictus*, Schneider) skin extract on U937 and K562 cells. Toxicon.

[27] Huang C, Chen A, Guo M, Yu J (2007). Membrane dielectric responses of bufalin-induced apoptosis in HL-60 cells detected by an electrorotation chip. Biotechnol Lett.

[28] Li D, Qu X, Hou K, Zhang Y, Dong Q, Teng Y, Zhang J, Liu Y (2009). PI3K/Akt is involved in bufalin-induced apoptosis in gastric cancer cells. Anticancer Drugs.

[29] Ko WS, Park TY, Park C, Kim YH, Yoon HJ, Lee SY, Hong SH, Choi BT, Lee YT, Choi YH (2005). Induction of apoptosis by Chan Su, a traditional Chinese medicine, in human bladder carcinoma T24 cells. Oncol Rep.

[30] Yun HR, Yoo HS, Shin DY, Hong SH, Kim JH, Cho CK, Choi YH (2009). Apoptosis induction of human lung carcinoma cells by Chan Su (*Venenum bufonis*) through activation of caspases. J Acupunct Meridian Stud.

[31] Ryu DS, Baek GO, Kim EY, Kim KH, Lee DS (2010). Effects of polysaccharides derived from Orostachys japonicus on induction of cell cycle arrest and apoptotic cell death in human colon cancer cells. BMB Rep.

[32] Yue T, Yin J, Li F, Li D, Du M (2010). High glucose induces differentiation and adipogenesis in porcine muscle satellite cells via mTOR. BMB Rep.

[33] Cho SY, Lee JH, Bae HD, Jeong EM, Jang GY, Kim CW, Shin DM, Jeon JH, Kim IG (2010). Transglutaminase 2 inhibits apoptosis induced by calcium-overload through down-regulation of Bax. Exp Mol Med.

[34] Lazebnik YA, Kaufmann SH, Desnoyers S, Poirier GG, Earnshaw WC (1994). Cleavage of poly(ADP-ribose) polymerase by a proteinase with properties like ICE. Nature.

[35] Fukuda K (1999). Apoptosis-associated cleavage of β-catenin in human colon cancer and rat hepatoma cells. Int J Biochem Cell Biol.

[36] De Graaf AO, De Witte T, Jansen JH (2004). Inhibitor of apoptosis proteins: new therapeutic targets in hematological cancer?. Leukemia.

[37] Jing Y, Watabe M, Hashimoto S, Nakajo S, Nakaya K (1994). Cell cycle arrest and protein kinase modulating effect of bufalin on human leukemia ML1 cells. Anticancer Res.

[38] Nasu K, Nishida M, Ueda T, Takai N, Bing S, Narahara H, Miyakawa I (2005). Bufalin induces apoptosis and the G0/G1 cell cycle arrest of endometriotic stromal cells: a promising agent for the treatment of endometriosis. Mol Hum Reprod.

[39] Takai N, Ueda T, Nishida M, Nasu K, Narahara H (2008). Bufalin induces growth inhibition, cell cycle arrest and apoptosis in human endometrial and ovarian cancer cells. Int J Mol Med.

[40] Choi JH, Choi AY, Yoon H, Choe W, Yoon KS, Ha J, Yeo EJ, Kang I (2010). Baicalein protects HT22 murine hippocampal neuronal cells against endoplasmic reticulum stress-induced apoptosis through inhibition of reactive oxygen species production and CHOP induction. Exp Mol Med.

[41] Murphy AN, Bredesen DE, Cortopassi G, Wang E, Fiskum G (1996). Bcl-2 potentiates the maximal calcium uptake capacity of neural cell mitochondria. Proc Natl Acad Sci USA.

[42] Thees S, Hubbard GB, Winckler J, Schultz C, Rami A (2005). Specific alteration of the Bax/Bcl2 ratio and cytochrome c without execution of apoptosis in the hippocampus of aged baboons. Restor Neurol Neurosci.

[43] Eckelman BP, Salvesen GS, Scott FL (2006). Human inhibitor of apoptosis proteins: why XIAP is the black sheep of the family. EMBO Rep.

[44] Hunter AM, LaCasse EC, Korneluk RG (2007). The inhibitors of apoptosis (IAPs) as cancer targets. Apoptosis.

[45] Galluzzi L, Larochette N, Zamzami N, Kroemer G (2006). Mitochondria as therapeutic targets for cancer chemotherapy. Oncogene.

[46] Gupta S, Kass GE, Szegezdi E, Joseph B (2009). The mitochondrial death pathway: a promising therapeutic target in diseases. J Cell Mol Med.

[47] Javadov S, Karmazyn M (2007). Mitochondrial permeability transition pore opening as an endpoint to initiate cell death and as a putative target for cardioprotection. Cell Physiol Biochem.

[48] Mook OR, Frederiks WM, van Noorden CJ (2004). The role of gelatinases in colorectal cancer progression and metastasis. Biochim Biophys Acta.

[49] Hazgui S, Bonnomet A, Nawrocki-Raby B, Milliot M, Terryn C, Cutrona J, Polette M, Birembaut P, Zahm JM (2008). Epigallocatechin-3-gallate (EGCG) inhibits the migratory behavior of tumor bronchial epithelial cells. Respir Res.

[50] Takada Y, Andreeff MM, Aggarwal BB (2005). Indole-3-carbinol suppresses NF-κB and IκBα kinase activation, causing inhibition of expression of NF-κB-regulated antiapoptotic and metastatic gene products and enhancement of apoptosis in myeloid and leukemia cells. Blood.

[51] Choi YH, Choi WY, Hong SH, Kim SO, Kim GY, Lee WH, Yoo YH (2009). Anti-invasive activity of sanguinarine through modulation of tight junctions and matrix metalloproteinase activities in MDA-MB-231 human breast carcinoma cells. Chem Biol Interact.

[52] Moon DO, Choi YH, Moon SK, Kim WJ, Kim GY (2010). Butein suppresses the expression of nuclear factor-κB-mediated matrix metalloproteinase-9 and vascular endothelial growth factor in prostate cancer cells. Toxicol In Vitro.

[53] Shin DY, Ryu CH, Lee WS, Kim DC, Kim SH, Hah YS, Lee SJ, Shin SC, Kang HS, Choi YH (2009). Induction of apoptosis and inhibition of invasion in human hepatoma cells by anthocyanins from meoru. Ann NY Acad Sci.

